# Maternal and perinatal characteristics of small-for-gestational-age newborns: Ten-year experience of a single center

**DOI:** 10.4274/jtgga.2016.0228

**Published:** 2017-06-01

**Authors:** Nihal Şahin Uysal, Çağrı Gülümser, Filiz Bilgin Yanık

**Affiliations:** 1 Department of Obstetrics and Gynecology, Division of Perinatology, Başkent University Ankara Hospital, Ankara, Turkey

**Keywords:** small for gestational age, risk factors, obstetric outcome

## Abstract

**Objective:**

To analyze the maternal and perinatal characteristics of small-for-gestational-age (SGA) newborns compared with appropriate-for-gestational-age (AGA) newborns in singleton pregnancies managed at our hospital between January 2006 and December 2015.

**Material and Methods:**

The study (n=456) and control (n=4925) groups included pregnancies resulting in SGA and AGA newborns, respectively. Additionally, two SGA subgroups were defined according to abnormal (n=34) and normal (n=57) Doppler findings. Maternal demographic features; intracytoplasmic sperm injection (ICSI) pregnancies; gestational age at delivery; birth weight; major congenital anomalies, karyotype abnormalities, and genetic syndromes; maternal and obstetric problems such as hypertensive disorders, diabetes, oligohydramnios, preterm birth; admission to the neonatal intensive care unit (NICU), and perinatal mortality were recorded, and the two groups were compared with respect to these parameters.

**Results:**

Mean maternal age, parity, gestational age at delivery, and birthweight were significantly lower; the frequencies of ICSI pregnancies, hypertensive disorders, oligohydramnios, preterm delivery, major congenital anomalies, karyotype abnormalities and genetic syndromes, admission to the NICU and perinatal mortality were significantly higher in the study group (p<0.05). None of the study parameters were significantly different between the two SGA subgroups (p>0.05).

**Conclusion:**

The association of SGA with ICSI pregnancies, hypertensive disorders, oligohydramnios, preterm delivery, congenital/chromosomal anomalies, NICU admission and perinatal mortality may be important in perinatal care. Clinical suspicion of SGA necessitates appropriate monitorization and management. Although obstetric outcomes were not significantly different between the two SGA subgroups with abnormal and normal Doppler findings in this study, this finding must be evaluated with caution due to the small sizes.

## INTRODUCTION

Small for gestational age (SGA) newborns are defined as birthweight <10^th^ percentile according to the gestational age (1). Although constitutional factors such as female sex, ethnicity, parity or maternal body mass index, might be the cause in most SGA newborns, various maternal, fetal or placental disorders may play role in the remainder. This latter group encompasses newborns with intrauterine growth restriction (IUGR), or in other words, fetal growth restriction (FGR) (2). Not all, but some cases of pathologic SGA may be differentiated from constitutional cases according to the presence of ultrasonographic findings such as declining fetal growth curve, oligohydramnios, abnormal Doppler indices, biometric measurements <3^rd^ percentile, as well as abnormal fetal anatomy (3). Nevertheless, several studies indicate that, SGA as a whole group, is associated with an increased risk of both neonatal morbidity, including respiratory distress syndrome, intraventricular hemorrhage, seizure, sepsis and perinatal mortality (4). Thus, the management of SGA fetuses and newborns differ from those that are appropriate for gestational age (AGA) due to the increased risk of perinatal and neonatal adverse outcomes. The effects of SGA are observed in the immediate neonatal period and in infancy, childhood, and adulthood. Long-term adverse outcomes such as neurocognitive impairment, obesity, type 2 diabetes mellitus, hypertension, and cardiovascular diseases are more frequently emphasized in recent studies (5, 6).

The aim of this study was to analyze the maternal and perinatal characteristics of small-for-gestational age-newborns compared with appropriate-for-gestational-age newborns in singleton pregnancies managed at our hospital between January 2006 and December 2015. The data obtained in this study may recall attention to the early suspicion and management of SGA in daily obstetric practice.

## MATERIAL AND METHODS

This study was approved by the Institutional Review Board and Ethics Committee of Başkent University. Başkent University Ankara Hospital delivery records and patients' files between January 2006 and December 2015 were retrospectively analyzed to determine SGA and AGA newborns. Only singletons were evaluated (n=5757) excluding twin and triplet pregnancies. SGA was defined as birthweight <10^th^ percentile and AGA was defined as birthweight between the 10^th^ and 90^th^ percentiles with respect to gestational age (7). The study group included 456 SGA newborns and the control group comprised 4925 AGA newborns. Maternal demographic features; intracytoplasmic sperm injection (ICSI) pregnancies; advanced maternal age; gestational age at delivery; birth weight; major congenital anomalies, karyotype abnormalities and genetic syndromes; maternal and obstetric problems such as hypertensive disorders, diabetes, oligohydramnios, preterm birth; admission to the neonatal intensive care unit (NICU), and perinatal mortality were recorded and the two groups were compared with respect to these parameters. Maternal age ≥35 years at birth was accepted as advanced maternal age. Chronic hypertension, gestational hypertension, preeclampsia, eclampsia, and superimposed preeclampsia were considered as maternal hypertensive disorders. Maternal diabetes included pregestational and gestational diabetes. When the deepest single pocket of amniotic fluid was measured as <2 cm at the second or third trimester ultrasonography, it was accepted as oligohydramnios. Preterm birth was defined as deliveries less than 37 gestational weeks. Perinatal mortality included intrauterine, intrapartum, and neonatal deaths within the postpartum first 28 days.

Doppler ultrasonography examinations of the study group including SGA newborns were also evaluated; however, we were not able to obtain the examination results of most of the cases because of the retrospective design of the study, and due to the renewal of the electronic recording system of the hospital. Thus, two subgroups were defined as abnormal and normal Doppler findings including 34 and 57 cases, respectively. Doppler examinations were accepted as abnormal when at least one of the following findings were present at any gestational age ≥24 weeks: umbilical artery (UA) S/D ratio >95^th^ percentile; pulsatility index (PI) >95^th^ percentile; absent or reversed end-diastolic flow (AREDF) in the UA; absent or reversed a-wave in ductus venosus (DV); and cerebroplacental ratio (CPR) was defined as middle cerebral artery (MCA) PI/UA PI <5^th^ percentile. The maternal and perinatal characteristics mentioned before were compared between the two SGA subgroups with abnormal and normal Doppler findings.

Statistical analysis was performed using SPSS for Windows, version 22.0. Mann-Whitney U, χ^2^, and Fisher’s exact tests were used where appropriate. A p value of <.05 was considered statistically significant.

## RESULTS

The frequency of the SGA newborns among singletons was 7.92% (456/5757) between January 2006 and December 2015 at Başkent University Hospital.

The mean maternal age, parity, gestational age at delivery and birth weight were significantly lower in the study group with SGA compared with the control group (30.61±5.25 vs. 31.11±4.63; 0.41±0.66 vs. 0.49±0.68; 37.01±3.42 vs. 38.04±2.37 and 2310.45±605.66 vs. 3222.91±502.11, respectively, (p<.05). There was no significant difference between the study and the control groups with respect to gravidity (p>.05) (Table 1).

When the two groups were compared with respect to risk factors for SGA and obstetric outcomes, it was observed that the rates of ICSI pregnancies, hypertensive disorders, oligohydramnios, preterm delivery, major congenital anomaly/chromosomal anomaly/syndrome were significantly higher in the study group (12.3% vs. 8%; 12.9% vs. 3.6%; 12.3% vs. 3.1%; 22.6% vs. 10.6% and 8.8% vs. 4.2%, respectively, p<.05). NICU admission and perinatal mortality were also significantly more frequent in the study group (26.7% vs. 8.4% and 5.3% vs. 1.2%, respectively, p<.05) (Table 2). Perinatal mortality was due to intrauterine exitus, anomaly-related mortality, and prematurity-related mortality in 25%, 33.3%, and 41.7% of cases in the study group, whereas it was 15%, 58.3%, and 26.7% in the control group, respectively. There were no significant differences between the study and control groups with respect to advanced maternal age and diabetes (p>.05) (Table 2).

In the subgroup analyses of SGA newborns with abnormal and normal Doppler findings, there were no significant differences in the mean maternal age, gravidity, parity, gestational age at delivery, and birth weight (p>.05) (Table 3).

The rates of risk factors and adverse obstetric outcomes, including ICSI pregnancies, hypertensive disorders, oligohydramnios, preterm delivery, major congenital anomaly/chromosomal anomaly/ syndrome, NICU admission, and perinatal mortality, did not significantly differ between the two SGA subgroups with abnormal and normal Doppler findings (p>.05) (Table 4).

## DISCUSSION

Whether the topic is ‘small for gestational age’ or ‘fetal growth restriction’ the complexity of the definitions must be kept in mind while reviewing the literature. SGA is accepted as birthweight <10^th^ percentile according to the gestational age using a population-based reference. The diagnosis of SGA would be more accurate if population-based birthweight standards were determined with healthy mothers without risk factors for FGR. Some studies in the literature used their own birthweight nomograms, whereas others, as in this study, used a different population-based nomogram (2, 4, 8). There are various single-center studies from Turkey that evaluated birthweight distribution, which probably cannot be generalized to the whole Turkish population (9, 10). Therefore, although it is a limitation of this study, we selected a population-based reference from the United States of America, which was determined to be the most powerful and most contemporary of the growth curves available (7).

The frequency of SGA newborns ranges widely from 3.5% to 17.9% in several studies in the literature due the different definitions and references used (2, 11). The rate of SGA newborns in our study appeared as 7.92%.

The current study aimed to compare the maternal and perinatal characteristics of the SGA newborns with those of AGA newborns in a single center within a ten-year period. By definition, the SGA group may include both constitutionally small but healthy newborns and those who are pathologically small; however, regarding the perinatal outcome, these two groups of SGA babies may differ from each other (12). On the other hand, the AGA group may also include both normal babies and babies who are unable to reach their biologic growth potential. Even though both SGA and AGA groups may include disordered babies, this proportion is expected to be much smaller in the AGA group, and reports in the literature agree in that perinatal morbidity and mortality rates in SGA newborns are higher (13, 14). Perinatal mortality was 5.3% (24/456) in the SGA group in our study, and it was significantly higher when compared with the 1.2% (60/4925) in the AGA group (Table 2). Perinatal morbidity such as sepsis, respiratory distress syndrome, bronchopulmonary dysplasia, intraventricular hemorrhage, necrotizing enterocolitis, retinopathy of prematurity, hypoxic ischemic encephalopathy, hypoglycemia, hyperbilirubinemia, and polycythemia might be observed more commonly in SGA newborns (15). In our retrospective data, we were not able to analyze the perinatal morbidity in the study and control groups in detail; however, the frequency of NICU admission was significantly higher in the SGA group, and this may indirectly reflect a higher morbidity rate.

Risk factors for SGA may be listed as follows: constitutionally small mothers; poor maternal nutrition; maternal and fetal infections; congenital malformations; chromosomal aneuploidies; inherited syndromes; tobacco, alcohol or illegal drug use; vascular disease; pregestational diabetes; chronic hypoxia; anemia; placental and cord abnormalities; and infertility (16).

In our study, the rates of ICSI pregnancies and major congenital anomalies/chromosomal anomalies/syndromes were significantly higher in the SGA group compared with the AGA group, in accordance with the literature (16, 17, 18). Zhu et al. (18) and Valenzuela-Alcaraz et al. (19) reported an increased incidence of SGA infants in women with a history of infertility with or without infertility treatment, and Valenzuela-Alcaraz et al. (19) also described a preferential association of SGA with treated infertility, either by ovulation induction or ICSI. We were not able to compare the study and control groups with respect to this parameter because the history of infertility was not recorded in our data; thus, we can only say that ICSI is a risk factor for SGA, either by itself or due to the history of infertility, not specified in our study.

Regarding maternal age, some studies defined young age and some defined advanced age as risk factors for SGA, whereas others failed to find any association (2). In our study, mean maternal age and parity were significantly lower in the SGA group and advanced maternal age did not appear to be a risk factor. It is generally accepted that nulliparity increases the risk of SGA infants when compared with multiparity, which may be considered as in accordance with our study (20, 21); however, conflicting results have also been reported (2).

In this study, the rate of hypertensive disorders was significantly higher in the SGA group compared with the AGA group, which is consistent with the results of various studies in the literature (22, 23, 24). Another comorbidity evaluated in our study was diabetes, including both pregestational and gestational diabetes, and the rate did not differ significantly between the study and control groups. In the literature, it is reported that diabetes with vasculopathy was associated with increased SGA (25). Also, Langer et al. (26) suggested that a relationship existed between the level of glycemic control and neonatal weight, which means that poor glycemic control is associated with large-for-gestational-age babies, whereas stringent glycemic control is associated with SGA babies.

The rate of oligohydramnios was significantly higher in the SGA group in our study; although we did not separately evaluate the subgroup with placental insufficiency as the underlying etiology, it is known that oligohydramnios mostly accompanies in this circumstance (27).

The preterm delivery rate in our study was significantly higher in the SGA group (22.6%) compared with the AGA group, although not classified as iatrogenic or spontaneous. It is well known that SGA is one of the most common indications for medical intervention resulting in preterm birth (28). FGR has also been recognized as a cause in spontaneous preterm labor and suggests a fetal role (29). Placental insufficiency and inadequate perfusion of the fetus might result in fetal distress and premature activation of the hypothalamo-pituitary-adrenal axis.

Abnormal Doppler indices may be useful to differentiate fetuses with pathologic growth restriction due to placental insufficiency, from those that are constitutionally small but normal. For subgroup analyses, we were able to document the Doppler studies of only 91 SGA newborns out of 456. When we analyzed the subgroups of SGA with abnormal (n=34) and normal (n=57) Doppler indices, there were no significant differences with respect to the study parameters, namely: maternal demographic features and rates of ICSI pregnancies, hypertensive disorders, oligohydramnios, preterm delivery, major congenital anomaly/chromosomal anomaly/syndrome, NICU admission, and perinatal mortality. Therefore, according to our results, abnormal Doppler findings were not significantly more common in SGA newborns with adverse outcomes such as preterm delivery, major congenital anomaly/chromosomal anomaly/syndrome, NICU admission or perinatal mortality. However, this finding must be evaluated with caution due to the small sizes of the subgroups. One other limitation of our study is that we were not able to analyze and compare the obstetric outcomes according to each Doppler abnormality separately, again because of the small sample size. Unterscheider et al. (30) reported that Doppler interrogation of the UA and MCA remained the most useful tool in identifying fetuses at risk of adverse perinatal outcome, capturing 88% of all adverse outcomes; however, management of SGA fetuses is more complex and Doppler indices, biophysical profile scoring, amniotic fluid volume, and maternal health status might be in consideration.

The present study could not give any results about specific morbidities and long-term health outcomes of SGA newborns. As we had to analyze the data retrospectively. We were not able to document and evaluate all risk factors including tobacco, alcohol or illegal drug use; previous SGA child; weight gain during pregnancy; body mass index; socioeconomic status, and numbers of prenatal visits.

In conclusion, the definition, management, and timing of delivery of SGA fetuses are still under debate. This study aimed to determine risk factors associated with SGA that might either be known preconceptionally or in the earlier weeks of gestation, or be recognized during antenatal follow-up, as well as the possible adverse outcomes awaiting SGA newborns. ICSI pregnancies and mothers with known hypertensive disorders might be followed-up more closely with respect to the increased risk of SGA. In the case of SGA, oligohydramnios, gestational hypertensive disorders, major congenital or chromosomal anomalies or syndromes can be more commonly encountered and these clinical conditions might already be present before the appearance of abnormal biometric measurements and Doppler findings, or might be diagnosed later. More detailed anatomic screening of fetuses with SGA may provide to identify the anomalies; on the other hand, oligohydramnios or hypertension may be clues for recognizing SGA. According to the results of our study, regarding perinatal outcomes of SGA newborns, preterm birth, NICU admission and perinatal mortality risks are increased; therefore, the delivery of these fetuses must be planned at appropriate centers. 

## Figures and Tables

**Table 1:**
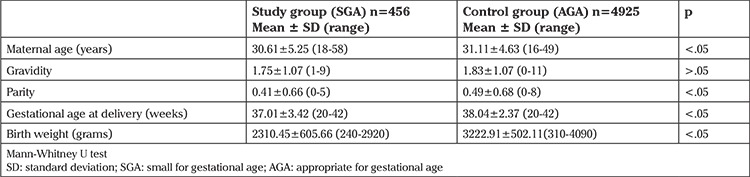
Demographic features of the study and control groups

**Table 2:**
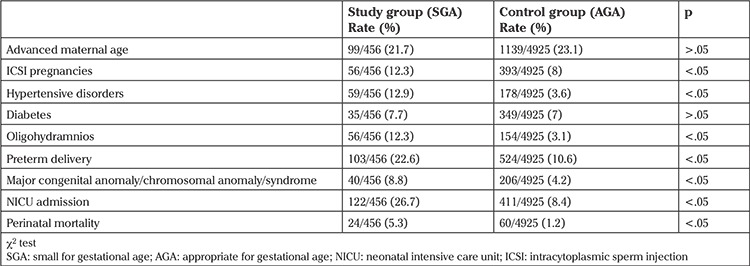
Risk factors for small-for-gestational-age newborns and obstetric outcomes in the study and control groups

**Table 3:**
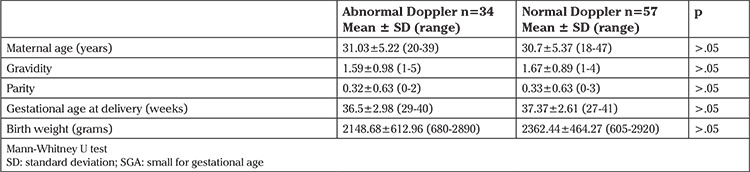
Demographic features of small-for-gestational-age newborns with abnormal and normal Doppler findings

**Table 4:**
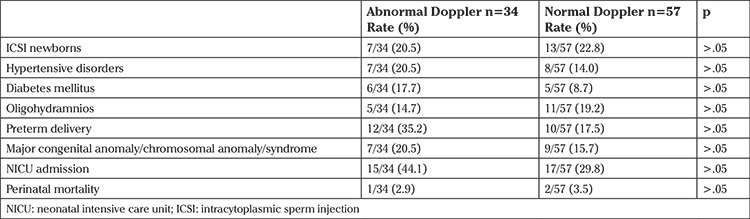
Risk factors and obstetric outcomes of the small-for-gestational-age newborns with abnormal and normal Doppler findings
